# Diversity of Pseudomonas aeruginosa Temperate Phages

**DOI:** 10.1128/msphere.01015-21

**Published:** 2022-02-23

**Authors:** Genevieve Johnson, Swarnali Banerjee, Catherine Putonti

**Affiliations:** a Bioinformatics Program, Loyola University Chicago, Chicago, Illinois, USA; b Department of Mathematics and Statistics, Loyola University Chicago, Chicago, Illinois, USA; c Department of Biology, Loyola University Chicago, Chicago, Illinois, USA; d Department of Microbiology and Immunology, Stritch School of Medicine, Loyola University Chicago, Maywood, Illinois, USA; University of Michigan-Ann Arbor

**Keywords:** *Pseudomonas aeruginosa*, prophages, temperate phages

## Abstract

Modern sequencing technologies have provided insight into the genetic diversity of numerous species, including the human pathogen Pseudomonas aeruginosa. Bacterial genomes often harbor bacteriophage genomes (prophages), which can account for upwards of 20% of the genome. Prior studies have found P. aeruginosa prophages that contribute to their host’s pathogenicity and fitness. These advantages come in many different forms, including the production of toxins, promotion of biofilm formation, and displacement of other P. aeruginosa strains. While several different genera and species of P. aeruginosa prophages have been studied, there has not been a comprehensive study of the overall diversity of P. aeruginosa-infecting prophages. Here, we present the results of just such an analysis. A total of 6,852 high-confidence prophages were identified from 5,383 P. aeruginosa genomes from strains isolated from the human body and other environments. In total, 3,201 unique prophage sequences were identified. While 53.1% of these prophage sequences displayed sequence similarity to publicly available phage genomes, novel and highly mosaic prophages were discovered. Among these prophages, there is extensive diversity, including diversity within the functionally conserved integrase and C repressor coding regions, two genes responsible for prophage entering and persisting through the lysogenic life cycle. Analysis of integrase, C repressor, and terminase coding regions revealed extensive reassortment among P. aeruginosa prophages. This catalog of P. aeruginosa prophages provides a resource for future studies into the evolution of the species.

**IMPORTANCE** Prophages play a critical role in the evolution of their host species and can also contribute to the virulence and fitness of pathogenic species. Here, we conducted a comprehensive investigation of prophage sequences from 5,383 publicly available Pseudomonas aeruginosa genomes from human as well as environmental isolates. We identified a diverse population of prophages, including tailed phages, inoviruses, and microviruses; 46.9% of the prophage sequences found share no significant sequence similarity with characterized phages, representing a vast array of novel P. aeruginosa-infecting phages. Our investigation into these prophages found substantial evidence of reassortment. In producing this, the first catalog of P. aeruginosa prophages, we uncovered both novel prophages as well as genetic content that have yet to be explored.

## INTRODUCTION

High-throughput short-read and long-read sequencing technologies have produced >300,000 prokaryotic genomes to date. Annotation and analysis of these genomes have found phage genes and prophages, which can be integrated into the bacterial genome or persist as an extrachromosomal plasmid. Early estimates found that prophages account for 10 to 20% of the host’s genome (1). The presence of a prophage within the bacterial genome can give the host a selective advantage (2, 3). Prophages can also protect the bacterial cell from environmental stressors and confer antibiotic resistance to the bacterial host (4). While some prophage sequences may persist only through the lysogenic life cycle, others, temperate phages, are able to also display the lytic life cycle. These temperate phages can contribute greatly to the wide genomic diversity of bacteriophages through horizontal gene transfer and recombination (5).

Prophage and phage sequence prediction tools, including Phage Finder (6), PHAST (7), PHASTER (8), Prophinder (9), and VirSorter (10), have been used to catalog prophage sequences from microbial communities (11, 12) and publicly available genomes of several bacterial taxa, e.g., Staphylococcus aureus (13), Salmonella, and Escherichia species (6, 9, 14–16), Mycobacterium smegmatis (17), Klebsiella pneumoniae (18), and Staphylococcus pneumoniae (19). The use of phage sequence prediction tools throughout these studies shows the high prevalence of prophages within a multitude of different bacterial genera. These studies have also shown that while there are several conserved gene regions, e.g., integrases and terminases, between phages that infect the same bacterial species, there is also distinct mosaicism that results in broad phage diversity.

While numerous obligately lytic phages have been described for Pseudomonas aeruginosa, research into P. aeruginosa prophages has primarily focused on those associated with increased bacterial pathogenicity and fitness. Prophage genes carried by P. aeruginosa can have a profound effect on bacterial phenotype, competitiveness, and pathogenicity (20). The Liverpool epidemic strain (LES) of P. aeruginosa, which has been associated with infections in cystic fibrosis patients, harbors 5 prophages: LESϕ2, LESϕ3, LESϕ4, LESϕ5, and LESϕ6 (21). Not only do the prophages improve the invasiveness of LES but they also assist in the displacement of other P. aeruginosa strains by LES, allowing LES full competitive advantage in lung infections (21). The P. aeruginosa phage FIZ15 causes lysogenic conversion in P. aeruginosa PAO1, leading to PAO1’s increased resistance to phagocytosis, increased resistance to normal human serum, and increased adhesion to human epithelial cells (22). P. aeruginosa prophages can also be responsible for producing cytotoxins, as is the case for the Pseudomonas φCTX phage (23). In yet another example, *Inoviridae* prophages, such as the Pf family of prophages, can promote biofilm formations in P. aeruginosa (24). The results of Knezevic et al. (25) suggest that Pf1-like prophages are abundant among P. aeruginosa strains.

While there have been a few studies focused on the diversity of specific types of P. aeruginosa phages, there has yet to be a comprehensive study on the diversity of prophages infectious to P. aeruginosa. Here, we conduct an investigation of all publicly available P. aeruginosa genomes, which now exceeds 5,383 genomes, with particular focus on temperate, tailed P. aeruginosa-infecting phages. Cataloging P. aeruginosa temperate phages serves two purposes. First, it provides insight into the diversity and putative roles that phages play in P. aeruginosa fitness and pathogenicity. Second, temperate phages can be viable candidates for phage therapy. Prior studies have explored P. aeruginosa phage use in animal models (26–30) as well as in humans (31–68).

## RESULTS

### Prophages of P. aeruginosa.

VirSorter identified 49,102 putative prophage sequences in the 5,383 publicly available P. aeruginosa genomes examined here (Table 1). These genomes represent isolates from various human body sites, the environment, and industrial settings. The isolation sites were significantly associated with the number of predicted prophage sequences for the strains of P. aeruginosa after normalization (*P* < 2.2e−16). Of the total predicted prophages, 6,852 prophages were of category 1 and category 4, the most confident categories for unintegrated and integrated prophages, respectively. Per VirSorter’s documentation, sequences predicted within these two categories have significant enrichment of virus-like genes and/or non-*Caudovirales* genes over the entire predicted region and encode at least one hallmark viral gene (10). The predicted prophage sequences can be found at http://doi.org/10.5281/zenodo.5072377. Given that these high-confidence predictions likely represent viable temperate phages, further analysis was restricted to category 1 and 4 prophage sequences only. A total of 3,672 P. aeruginosa genomes encoded prophage sequences in these categories. While these genomes had on average 1 or 2 prophages, P. aeruginosa XDR-PA (GCA_900707735.1) harbored 15 predicted prophage sequences.

**TABLE 1:** Summary statistics of VirSorter prophage prediction results

No. of prophages predicted for category:					
1	2	3	4	5	6
2,668	12,579	5,666	4,184	17,367	6,638

Categories 1 and 4 are the highest-confidence predictions. Categories 3 and 6 are the lowest-confidence predictions, e.g., typically partial phages or phage-like genes.

### Genetic diversity of P. aeruginosa prophages.

The 6,852 category 1 and 4 predicted prophages were clustered based on sequence homology in an effort to ascertain the number of unique prophages that were identified. In total, there are 3,201 unique clusters of prophages. The largest cluster contains 169 phages; 2,496 of these clusters contain only 1 phage, meaning that the predicted prophage sequence was not detected in any of the other P. aeruginosa genomes examined (see Fig. S1A in the supplemental material). Similar prophage sequences, i.e., prophage sequences belonging to the same cluster, can be the result of (i) pervasive phages or (ii) genomes from the same strain and/or clonal strains. The genomic diversity of P. aeruginosa genomes harboring prophages belonging to the same cluster were examined further using average nucleotide identity (ANI) as a means of assessing genomic similarity. For the largest cluster, the P. aeruginosa strains had an ANI of 99.86% (Fig. S1B). The second largest cluster, *n* = 126, had an ANI of 99.00% (Fig. S1C). Neither of these clusters included genome sequences with a pairwise ANI of 100%. Nevertheless, the lack of complete metadata for publicly available genomes limits our ability to distinguish between the two scenarios and, thus, the ability to consider pervasiveness of prophage species.

10.1128/msphere.01015-21.1 FIG S1 Examination of unique prophage sequences. (A) Distribution of the size of prophage clusters based on prophage sequence similarity. (B) P. aeruginosa genome assembly similarity (ANI) for strains containing prophages from the largest identified cluster (169 prophage sequences). (C) P. aeruginosa genome assembly similarity (ANI) for strains containing prophages from the second largest identified cluster (126 prophage sequences). Download FIG S1, PDF file, 0.2 MB. Copyright © 2022 Johnson et al. 2022 Johnson et al. https://creativecommons.org/licenses/by/4.0/ This content is distributed under the terms of the Creative Commons Attribution 4.0 International license.

Next, the taxonomy of the 6,852 prophages was determined by querying each predicted prophage sequence against all publicly available characterized, sequenced phages. This resulted in 11 classified as *Inoviridae*, 106 as *Microviridae*, 672 as *Myoviridae*, 105 as *Podoviridae*, and 2,744 as *Siphoviridae*. The remaining 3,214 prophages did not share significant sequence similarity to any characterized, sequenced phages; thus, a taxonomic classification could not be made.

**FIG 1:**
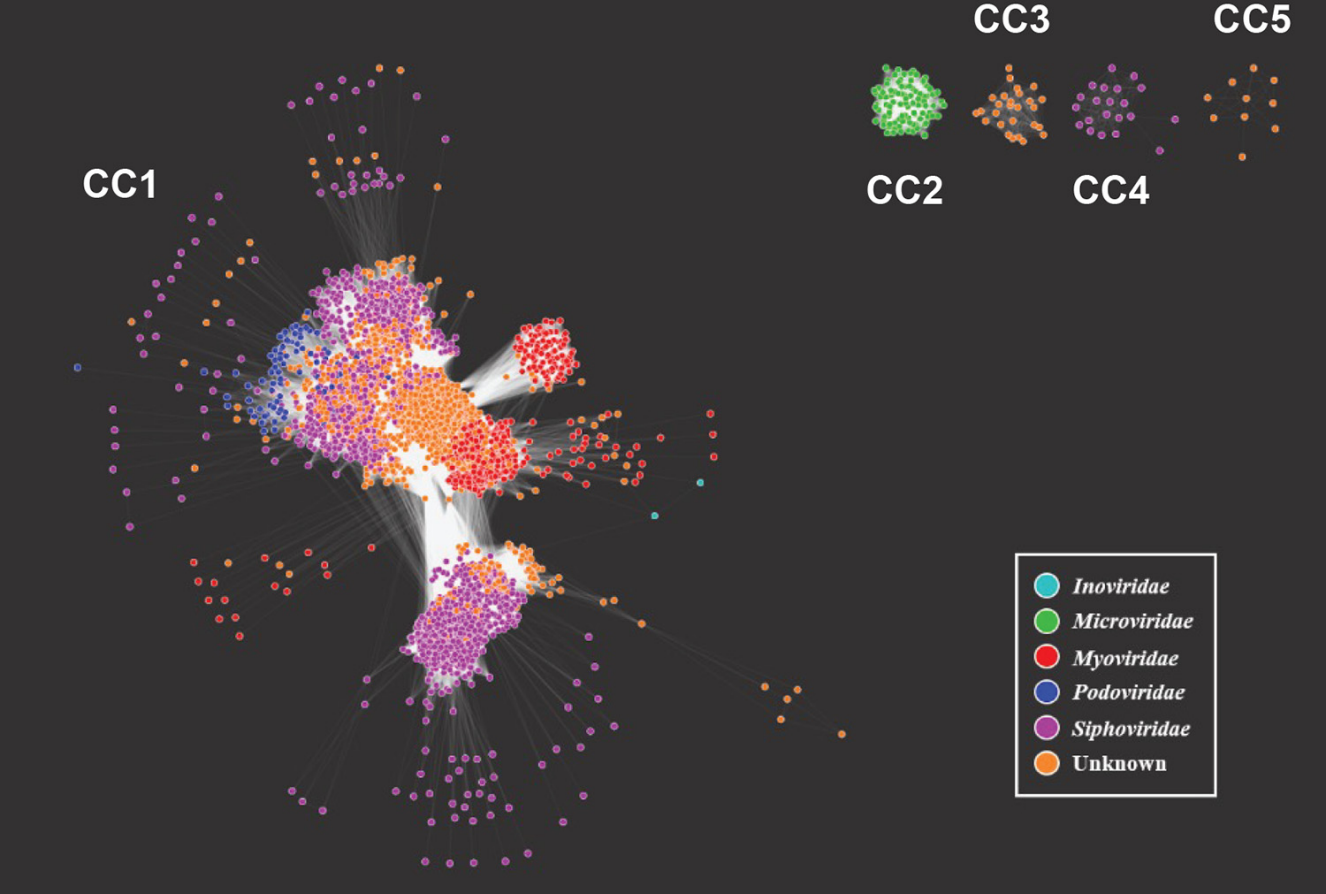
Network of P. aeruginosa predicted prophages. Prophages are the nodes color-coded by taxonomy: *Inoviridae*, *Microviridae*, *Myoviridae*, *Podoviridae*, *Siphoviridae*, or unknown. Prophages are connected by the number of shared genes between each prophage. Five separate connected components (CC) represent these prophages, labeled CC1 through CC5.

The diversity of P. aeruginosa prophages was visualized through a network consisting of the predicted prophages as nodes and the connecting edges representative of the number of genes shared between each prophage. Further investigation into the category 1 and 4 prophage sequences revealed high-confidence predictions of sequences too small to be a viable phage. In an effort to focus our investigation on complete (and likely viable) prophages, we introduced a threshold; only edges representative of 5 or more shared genes between prophages (nodes) were retained; nodes that were not connected to any other node were removed from further consideration. After this thresholding, 6,676 of the originally predicted 6,852 (97.43%) prophages remained (Fig. 1). The network contained a total of 3,814,212 edges representing the genes shared between the prophages. It contains 2 *Inoviridae* prophages, 105 *Microviridae* prophages, 635 *Myoviridae* prophages, 99 *Podoviridae* prophages, 2,662 *Siphoviridae* prophages, and 3,173 prophages for which no taxonomic classification could be made (unknown).

The prophages cluster within 5 connected components (CC), labeled CC1, CC2, CC3, CC4, and CC5 in Fig. 1. The majority (6,513 prophages; 97.56%) belonged to one large connected component, CC1, which includes tailed prophages (*Myoviridae*, *Podoviridae*, and *Siphoviridae*), the *Inoviridae* prophages, and unclassified (unknown) prophages. The 105 *Microviridae* prophages (green nodes in Fig. 1) belong to their own connected component, CC2, sharing genes only among other microviruses. CC4 in Fig. 1 contains 21 *Siphoviridae* prophages, which are distinctly different from other *Siphoviridae* prophage sequences within the largest connected component. These prophage sequences were identified from 18 different P. aeruginosa genomes, derived from 3 different studies. Further investigation of these sequences revealed genetic homology to strains of Escherichia coli as well as to the E. coli lambda phage (Table S1).

10.1128/msphere.01015-21.3 TABLE S1 CC4 *Siphoviridae* prophages sequence homology to Lambdaviruses. Download Table S1, XLSX file, 0.01 MB. Copyright © 2022 Johnson et al. 2022 Johnson et al. https://creativecommons.org/licenses/by/4.0/ This content is distributed under the terms of the Creative Commons Attribution 4.0 International license.

CC3 and CC5 contained prophages classified as unknown (orange nodes in Fig. 1). The first of the unknown clusters consisted of 27 prophages predicted from 27 different genomes of P. aeruginosa. While BLAST queries of these prophage sequences revealed homology to a pyocin gene fragment (L06240.1), BAGEL4 (69), a bacteriocin prediction tool, did not predict these sequences to be bacteriocins. The other cluster classified as unknown contained 10 prophages predicted from 7 different P. aeruginosa genomes. The prophage sequences were input to BLAST and all found to have high similarity (over 75%) to at least 1 of 2 uncultured *Caudovirales* phages (clone 3S_15, MF417945.1; clone 3S_19, MF417971.1), both identified through a skin metavirome project. Five of these predicted prophages were also similar (>70% identity and 8 to 24% query coverage) to the partial genomes of 2 *Myoviridae* isolates (isolate ctQcn1, BK028718.1; isolate ct2Fx3, BK037698.1). Further investigation of this cluster is required to make a taxonomic classification.

In a prior study, Pf1-like phages (*Inoviridae*) were found to be prevalent in P. aeruginosa strains, with ∼60% of 241 strains screened via PCR to contain at least 1 Pf1-like genetic element (25). Thus, we expected to find many more inoviruses in the 5,383 genomes examined. Prior studies have documented that current prophage prediction tools, including VirSorter, frequently miss inoviruses (10, 70, 71). Pf1-like genetic elements were identified in an additional 93 predicted prophages of lower-confidence VirSorter categories. These sequences had a high sequence similarity (>75% identity), but identities were only between one or a few genes (query coverage averaged 20%). Thus, we specifically mined the 5,383 P. aeruginosa genomes for Pf1, identifying the prophage (sequence identity and query coverage of >99%) in 123 of the genomes (Table S2); P. aeruginosa strain PSE6684 (GenBank accession no. CP053917.1) was predicted to contain 2 complete Pf1 prophages. Thus, VirSorter missed most instances of this well-studied prophage. Given this result, we reframe our catalog to be a representation of the tailed phages within publicly available P. aeruginosa strains.

10.1128/msphere.01015-21.4 TABLE S2 BLAST search for Pf1 prophage sequence in P. aeruginosa genomes. Query coverage refers to the percentage of the Pf1 prophage sequence (GenBank accession no. AY324828.1) that aligned to the subsequence of the P. aeruginosa genome assembly. Download Table S2, XLSX file, 0.01 MB. Copyright © 2022 Johnson et al. 2022 Johnson et al. https://creativecommons.org/licenses/by/4.0/ This content is distributed under the terms of the Creative Commons Attribution 4.0 International license.

### Diversity of P. aeruginosa prophage integrases, C repressors, and terminase coding sequences.

The associations between prophages that share homologous terminases and also share homologous integrases or C repressors were considered to investigate the genetic reassortment between prophage genomes that are likely to be temperate. Terminases are genes typically conserved among tailed phages. Prophage sequence annotation identified 283,493 coding regions within the 6,852 predicted prophage sequences; 14,172 of these coding regions are unique. Among these coding regions, 1,358 were annotated as integrases, 2,344 as C repressors, and 4,546 as terminases. To ensure that all integrase, C repressor, and terminase genes within these prophage sequences were included in our analysis, we conducted additional BLAST homology searches for representatives of these 3 genes. These queries identified an additional 12, 17, and 24 integrase, C repressor, and terminase coding regions, respectively. Thus, a total of 1,370 integrases, 2,361 C repressors, and 4,570 terminases are encoded within the predicted P. aeruginosa prophage sequences. These three marker genes were identified in 19.99%, 34.46%, and 66.34% of the predicted prophage sequences, respectively. A total of 850 prophages encoded both a terminase and an integrase, 391 prophages encoded both a terminase and a C repressor, and 713 prophages contained coding regions for all 3 of these genes (Fig. S2).

10.1128/msphere.01015-21.2 FIG S2 Phylogenetic tree of all annotated terminase coding regions. Average pairwise identity of 8.9% (*n* = 4,570). Terminases are color-coded by shared phage membership with integrase and/or C repressor homologous clusters. Download FIG S2, PDF file, 0.6 MB. Copyright © 2022 Johnson et al. 2022 Johnson et al. https://creativecommons.org/licenses/by/4.0/ This content is distributed under the terms of the Creative Commons Attribution 4.0 International license.

**FIG 2:**
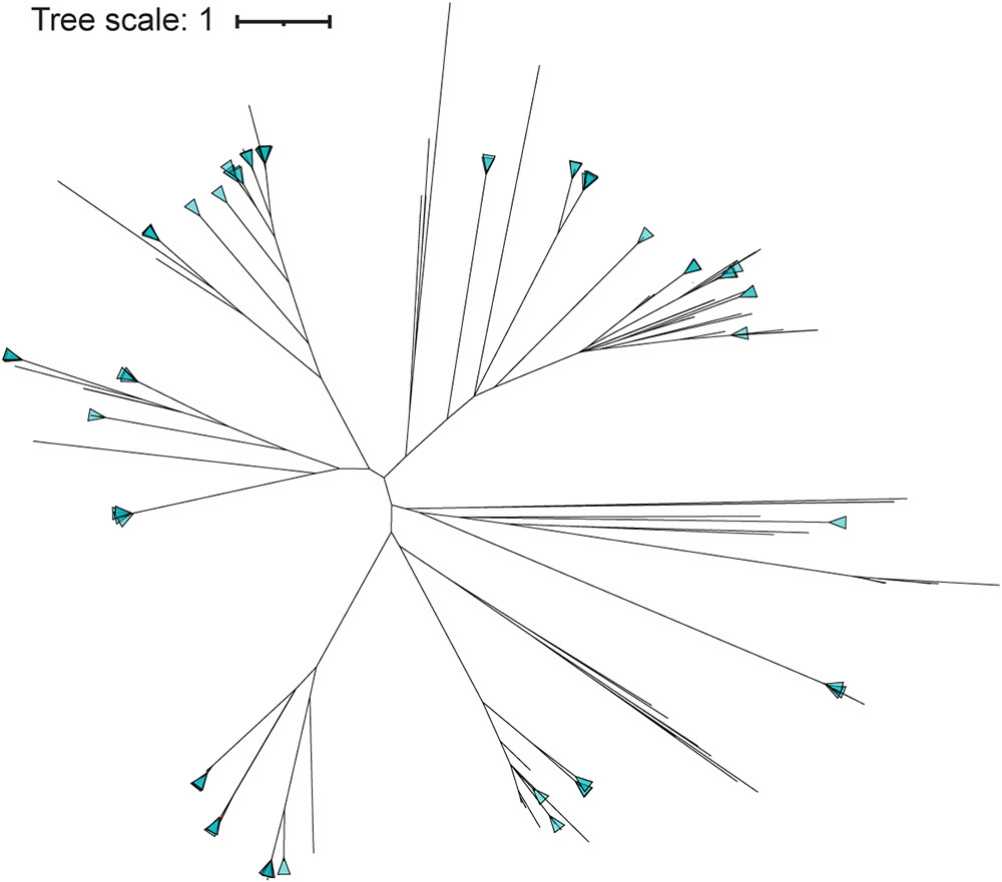
Phylogenetic tree of all annotated integrase coding regions.

**FIG 3:**
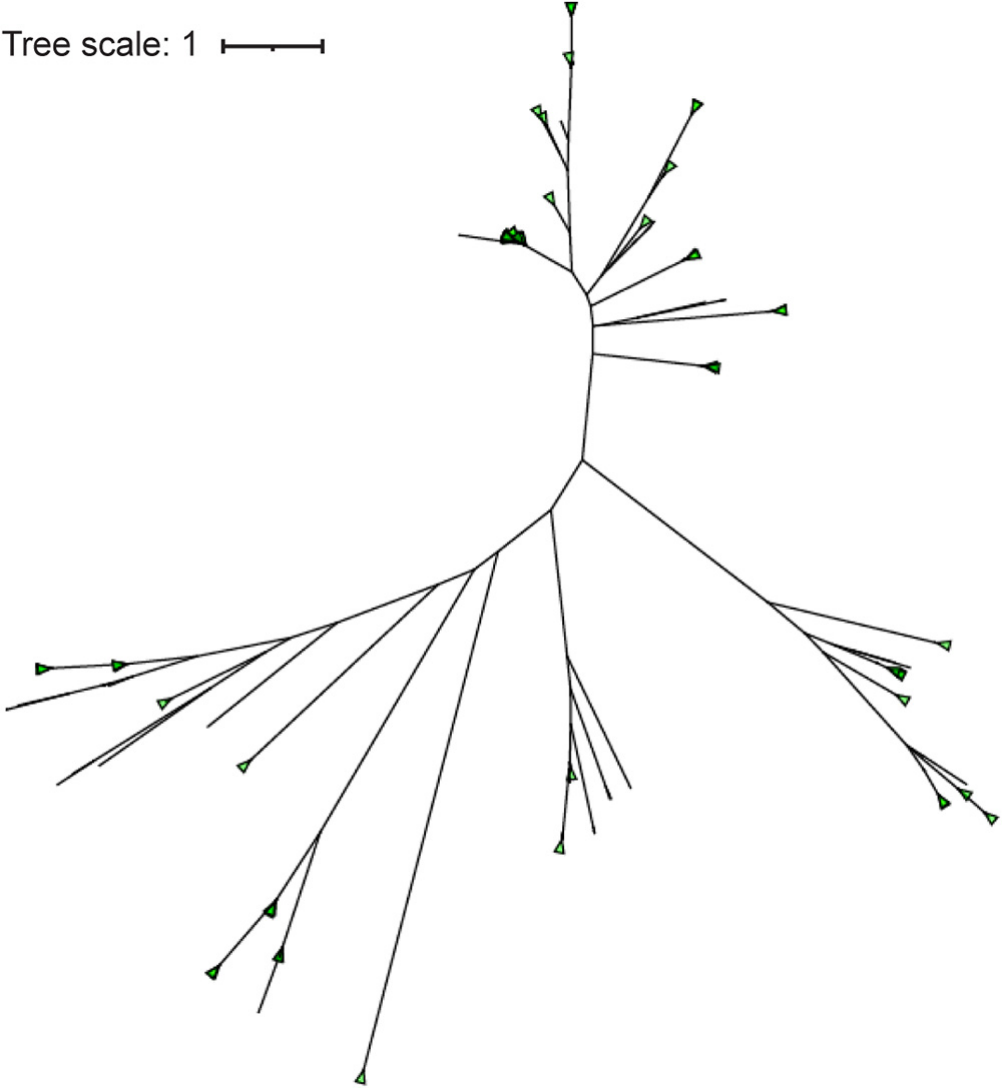
Phylogenetic tree of all annotated C repressor coding regions.

To investigate the diversity of integrase and C repressor sequences within the P. aeruginosa prophages, phylogenetic trees were derived. As Fig. 2 shows, there are distinct lineages of integrase genes among the prophages. Furthermore, these lineages were divergent, with an average amino acid pairwise identity of 16.1% (*n* = 1,370). We also examined the sequence diversity of the 2,361 C repressor coding sequences within the predicted P. aeruginosa prophages (Fig. 3). While distinct lineages were also observed, the set of C repressor sequences exhibited greater sequence similarity than the integrases; the average amino acid pairwise identity is 58.0%.

We next performed clustering of the integrase (*n* = 1,370), C repressor (*n* = 2,361), and terminase (*n* = 4,570) coding sequences. In total, 72 different clusters of integrases were identified, with the largest containing 110 integrase coding regions. The integrase sequences within this cluster exhibit little sequence variation (average pairwise identity, 98.3%). The C repressor sequences clustered into just 44 groups, with over half (*n* = 1,604; 67.94%) belonging to a single group. This largest cluster of C repressor coding regions contains an average pairwise identity of 99.7%. The 4,570 terminase coding regions clustered into 81 homologous groups. The largest of the terminase clusters contained 424 terminase coding regions with an average pairwise identity of 89.6%.

**FIG 4:**
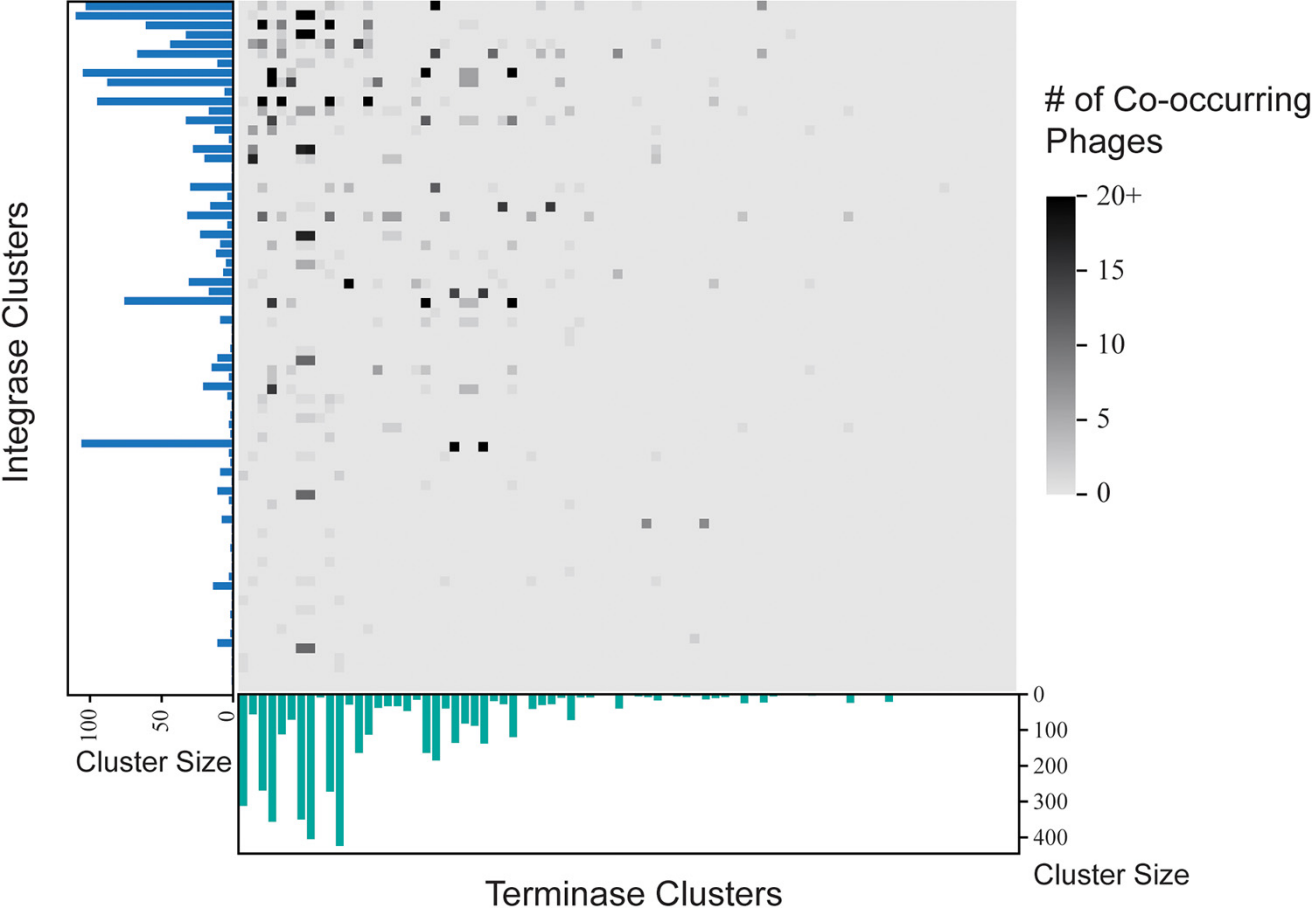
Prophage membership shared between integrase clusters and terminase clusters. The largest terminase clusters tend to share the most prophages with the largest integrase clusters.

Each prophage was next associated with the integrase, C repressor, and terminase clusters identified in the previous section. Between all 72 integrase clusters and all 81 terminase clusters, 40 integrase clusters shared all of their prophages with individual terminase clusters and 3 terminase clusters shared all of their prophages with individual integrase clusters. Figure 4 displays the distribution of shared prophages between the integrase clusters and the terminase clusters, with the highest numbers of cooccurring prophages existing between the largest integrase clusters and the largest terminase clusters.

**FIG 5:**
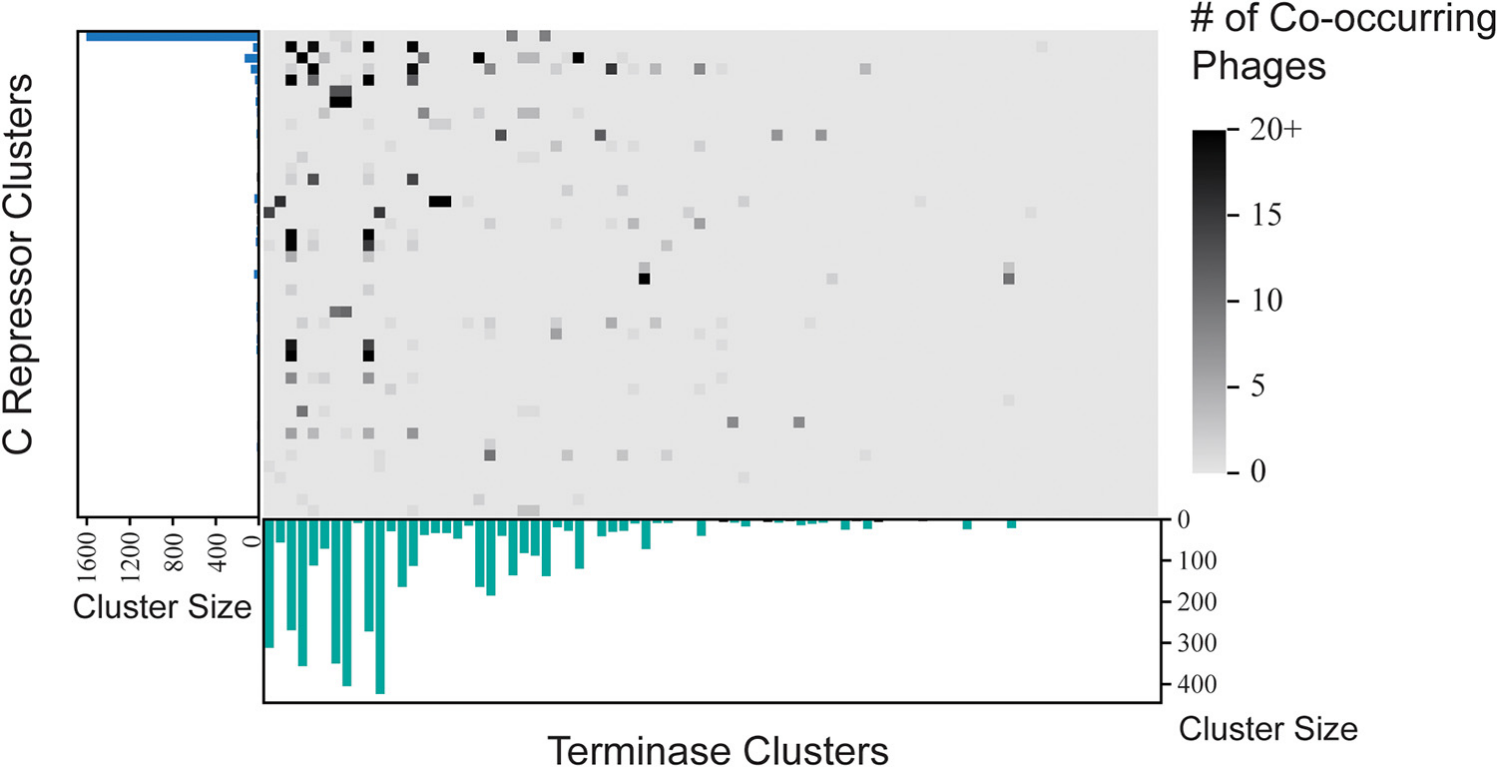
Prophage membership shared between C repressor clusters and terminase clusters. The largest terminase clusters tend to share the most prophages with the largest C repressor clusters.

Of the 44 C repressor clusters and the 81 terminase clusters, 17 C repressor clusters shared all of their prophages with individual terminase clusters and 5 terminase clusters shared all of their prophages with individual C repressor clusters. The C repressor clusters and the terminase clusters in Fig. 5 display a deduction similar to that shown in Fig. 4, wherein the most shared prophages are between the largest C repressor clusters and the largest terminase clusters.

The integrase cluster membership of a phage is not associated with the terminase cluster membership of the phage; phages within terminase clusters contain integrases from significantly more than 1 integrase cluster type (*P* = 8.275e−06). Similarly, the C repressor cluster membership of a phage is not associated with the terminase cluster membership of the phage; phages within terminase clusters contain C repressors from significantly more than 1 C repressor cluster (*P* = 8.079e−04).

### Antibiotic resistance, virulence factors, and metabolic genes encoded by prophages.

All category 1 and 4 predicted prophage sequences were examined for antibiotic resistance genes; 71 predicted antibiotic resistance genes from 42 different prophage genomes were identified (Table S3). The prophage sequences were next screened for virulence factors; 502 prophages possessed a total of 515 predicted virulence factor sequences (Table S4). The 515 predicted sequences were composed of 72 unique virulence factor sequences for 63 unique virulence genes, and 41 prophages contained both predicted virulence factors and predicted antibiotic resistance genes.

10.1128/msphere.01015-21.5 TABLE S3 Antibiotic genes identified in predicted prophage sequences. Download Table S3, XLSX file, 0.01 MB. Copyright © 2022 Johnson et al. 2022 Johnson et al. https://creativecommons.org/licenses/by/4.0/ This content is distributed under the terms of the Creative Commons Attribution 4.0 International license.

10.1128/msphere.01015-21.6 TABLE S4 Virulence factor genes identified in predicted prophage sequences. Download Table S4, XLSX file, 0.04 MB. Copyright © 2022 Johnson et al. 2022 Johnson et al. https://creativecommons.org/licenses/by/4.0/ This content is distributed under the terms of the Creative Commons Attribution 4.0 International license.

Next, all category 1 and 4 predicted prophage sequences were examined for the functional categories of their gene content. Per COG classification, 2,686 unique prophage genes that are classified as metabolic genes and 331 COGs associated with metabolism were identified (Table S5). The most frequently detected putative auxiliary metabolite genes are associated with amino acid metabolism, membrane transport, methane metabolism, and carbohydrate metabolism. Auxiliary metabolite genes for metal resistance and transport were also identified (Table S5).

10.1128/msphere.01015-21.7 TABLE S5 Prophage-encoded metabolite genes. Download Table S5, XLSX file, 0.02 MB. Copyright © 2022 Johnson et al. 2022 Johnson et al. https://creativecommons.org/licenses/by/4.0/ This content is distributed under the terms of the Creative Commons Attribution 4.0 International license.

## DISCUSSION

This is the first known report of the diversity of prophages among P. aeruginosa strains. We limited our analysis to only the highest category prophage predictions from VirSorter, categories 1 and 4. Other investigations of phages in genomic and metagenomic data sets have taken a less conservative approach, including VirSorter category 1, 2, 4, and 5 predictions (11, 70, 72). While others have noted that the lower confidence categories (3 and 6) tend to include only partial prophage genomes (11, 70, 72), our own prior work predicting prophages in the urinary microbiome found that most (∼71%) of the phage sequences in the category 2 and 5 predictions are not complete prophage sequences (11). While some nontailed phages were identified in the category 1 and 4 predictions, our direct search for the inovirus Pf1 (i) revealed many Pf1 prophages that were not detected, (ii) supports current literature acknowledging the limitations of current prophage prediction tools in identifying nontailed prophages, including inoviruses (10, 70, 71), and (iii) led to our focus on P. aeruginosa-infecting tailed prophages. Even with the stringent threshold applied, there are likely some false positives and prophage artifacts (nonfunctional prophages) included in these higher confidence predictions. For instance, 332 prophage predictions were less than 3,000 bp long, which is less than the smallest characterized Pseudomonas phage genome, PRR1 (NC_0082941), at 3,573 bp. These smaller predicted prophage sequences may be the result of erroneous calls by VirSorter or a result of fragmented bacterial assemblies, as most of the genomes examined here are draft assemblies.

The catalog of P. aeruginosa prophages includes 6,852 prophages from 5,383 genomes that were isolated from a variety of sources. We recognize that the genomes examined may include representatives of the same clonal strain. The lack of detailed metadata presents a challenge in ascertaining the source of many of these strains. Additionally, the core genome of the species is large and highly conserved. As prior comparative genomics studies of P. aeruginosa genomes found, strains belonging to the same group, which includes isolates from different sources (e.g., human and environmental), locations, and times, have ANI values of >99% (73–75). Thus, we cannot speculate as to the prevalence of the identified prophage species. By clustering the prophage sequences, however, we gain a glimpse into the diversity of prophages for the species, finding 3,201 unique prophage sequences. Because most of these clusters contain a single prophage sequence, we conclude that the genetic diversity of P. aeruginosa phages has yet to be fully explored.

In total, 68.21% of the genomes examined contained at least one category 1 or 4 prophage prediction. Lower confidence prophage sequences were far more abundant (Table 1). These regions may signify past phage infections. Prophages are believed to play a significant role in the evolution of this pathogen (76). On average, strains harbored only one or two prophage sequences. These prophages may inhibit additional prophage acquisition via superinfection exclusion. Prior studies of P. aeruginosa lysogens have found high rates of resistance to phage infection through prophage-mediated effects on structures required for phage attachment and/or adsorption (77, 78).

A level of 53.1% of the predicted prophages exhibited a query coverage greater than 50% and a percent identity of over 70% to publicly available phage genomes. These similarities were most frequent to tailed phages, which is expected given the tool used and the fact of the overrepresentation of tailed phages in sequence databases. Based upon sequence similarity, we have classified the majority of the predicted prophages as siphoviruses (*n* = 2,744). Most predicted prophage sequences (*n* = 3,214) did not meet our threshold of sequence similarity to characterized phages and are representative of either novel prophages or highly mosaic prophages infectious of P. aeruginosa. The query coverage threshold of 50% enabled taxonomic classification of mosaic prophages that share most of their genes with previously characterized phages. However, novel prophages or highly mosaic prophages were likely classified as unknown. Prior studies have observed mosaicism within P. aeruginosa*-*infecting siphoviruses (79). The network analysis performed here provides a means to predict the putative taxonomic classification of many of these unknown prophages based upon their proximity to classified prophages. As Fig. 1 shows, the majority of the unknown prophages clustered with siphoviruses or myoviruses. Nodes in the periphery of the large connected component CC1 share few genes with other predicted prophages and, thus, may represent families of phages yet to be characterized. The prophage sequences in CC4 include predictions from clinical isolates. Eight of these sequences are full-length lambda phage sequences; the remaining predicted prophages in this connected component are small sequences, partial representatives of the lambda phage genome (see Table S1 in the supplemental material). A lambda-like phage has recently been described for P. aeruginosa (80).

Further evidence of reassortment among P. aeruginosa infecting phages can be seen from our analysis of the terminases, integrases, and C repressor gene sequences. No association between prophages that share homologous terminases with prophages that share homologous integrases or C repressors was observed (Fig. 4 and 5). On average, for a given terminase group, these prophage sequences span 3 different integrase clusters. The same applies to C repressors where, on average, phages with a terminase also contain C repressors from approximately 2 different C repressor clusters.

The integrase and C repressor coding regions that were identified in the predicted prophages exhibited extensive diversity. The integrase coding regions shared an average amino acid pairwise identity of only 16.1%. This is far less than that seen for other pathogenic species, e.g., Staphylococcus aureus integrase diversity is a minimum of 38% nucleotide identity (13). When clustered based on sequence similarity, the integrases formed 72 distinct groups with very high similarity (>95% average pairwise identity) in the largest (*n* = 110) of the groups. Integrase diversity has also been observed for other pathogenic species, e.g., S. aureus has 8 major prophage integrase types (13), group B Streptococcus has 16 prophage integrase types (81), and Salmonella enterica has 23 integrase types among food-associated strains (15). In contrast to the integrases, less sequence diversity was observed among the P. aeruginosa prophage C repressor coding regions (average pairwise identity of 58.0%). These C repressor sequences clustered into 44 separate groups with high similarity (>86% average pairwise identity) within the largest (*n* = 1,604) of the groups. While the integrase gene and the C repressor gene are widely conserved in temperate phages, they display substantial diversity between their genomic groups.

Prior studies have found that moron (accessory) prophage genes can affect, e.g., biofilm formation, motility, and virulence factor production (see the review in reference 82). Furthermore, within P. aeruginosa the effects of the same moron gene can vary between strains (20). The survey of P. aeruginosa prophages here found a large reservoir of genes; over 14,000 unique coding regions were encoded by these prophages. Prior pangenome analysis of 1,311 P. aeruginosa genomes found that 8% of all genes unique to the bacterial genomes were phage genes (75). While most of the prophage sequences examined here do not encode antibiotic resistance genes or common virulence factors, many auxiliary metabolic genes were identified. Phage-encoded metabolic genes have been best studied in marine phages (83, 84), and it was more recently explored in groundwater (85). Prior investigation of a few lytic P. aeruginosa-infecting phages found 3% of genes to be putative auxiliary metabolic genes, and phage-specific metabolic effects during infection have been observed (86). Further investigation is needed to explore their putative role as well as the function of the numerous hypothetical proteins identified within the prophage sequences.

As previously mentioned, the phages identified here may not be an exhaustive list of the prophages harbored by the P. aeruginosa strains examined. VirSorter v.1 is optimized to recognize double-stranded DNA tailed phages (*Caudovirales*). These are the prophages that have been focused on here through our subsequent analysis of integrase, C repressor, and terminase gene sequences. Several different tools have been developed specifically for prophage identification, e.g., ProphET (87), PHASTER (8), and PhiSpy (88). Other tools, such as MARVEL (89), VIBRANT (90), VirFinder (91), DeepVirFinder (92), and VironFinder (93), have been optimized for viral sequence detection in metagenomic data. More recently, although after we began the study presented here, VirSorter2 (94) was released, expanding its classifiers to include *Caudovirales* as well as other viral groups. These other viral groups are not as well studied as *Caudovirales*, and future experimental work will be pivotal in increasing not only our knowledge of these phages but also genetic signatures that can be used to improve and/or validate bioinformatic predictions.

Beyond providing insight into the evolution of P. aeruginosa-infecting phages and P. aeruginosa strains, the prophages found in this study create a catalog of phages for potential use as phage therapies. Our analysis suggests that many of the high-confidence phages are temperate phages. While past phage therapies of P. aeruginosa infections have traditionally employed obligately lytic phages (31–68), temperate phages are also effective treatments when used in combination with other temperate phages as a cocktail. A 2015 study displayed successful results of temperate phage cocktails against strains of P. aeruginosa as well as against Clostridium difficile strains (95–97). Induction and characterization of temperate prophages provides additional resources in the face of growing antibiotic resistance. More importantly, the catalog presented here initiates exploration of the diversity of P. aeruginosa-infecting phages, the genetic content that they carry, and their impact on the fitness of the bacterial host.

## MATERIALS AND METHODS

### Cataloging P. aeruginosa prophages.

Through the Genome Information by Organism section of the NCBI Genome database, P. aeruginosa bacterial genomic assemblies were downloaded (September 2020). Genome quality was assessed, using PATRIC’s “Genome Quality” assessment (98) and CheckM (99) completeness and contamination scores through PATRIC. The following CheckM threshold was applied: completeness of >95% and contamination of <5%. In total, 5,383 genomes passed this threshold. All genomes were then entered into VirSorter v.1, a bioinformatic tool to predict phage sequences found inside bacterial genomes (10). VirSorter v.1 detects viral signals using both reference-based homology as well as reference-independent methods. It then predicts phage sequences with confidence levels ranging from 1 to 3 for extrachromosomal phages, e.g., lytic or plasmid phages, and 4 to 6 for prophages, where 1 and 4 are the highest confidence predictions and 3 and 6 are the lowest confidence predictions.

Identified prophage sequences were clustered by similarity using MeshClust (v.1.2.0) with a nucleotide sequence identity threshold of 0.95 (100). The largest clusters of prophages were further investigated. The bacterial sequences that the prophages were predicted from were compared using average nucleotide identity (ANI) to determine if the highly similar prophages were predicted from similar or dissimilar strains of P. aeruginosa. The software tool used to compute ANI is pyANI (v.0.2.11) (101).

### Taxonomic classification of P. aeruginosa prophages.

All category 1 and 4 sequences were compared to previously characterized phage genomes in an effort to determine their likely taxonomic family. Each sequence was queried against all complete and partial genome sequences in GenBank (organism “Virus,” division “PHG”) using local BLAST (102). This database includes 26,381 sequences, and the blastn algorithm was used. Homologous results with a query coverage greater than 50% and a percent identity over 70% were considered to be acceptable, and the taxonomies of the resulting similar phages were used to predict the taxonomy of the query phages.

### Evaluating genomic relationships between P. aeruginosa prophages.

Predicted phage sequences were then examined using Anvi’o v.6.2 to find the number of shared genes between each predicted phage (103). Anvi’o was used to identify homologs given the following parameters: MCL inflation value of 2 during cluster identification and a minbit heuristic score of 0.35 to remove weak gene matches. A Python script was used to produce an edgelist of each node’s connections to other nodes and the number of genes shared between the connected nodes. The edgelist file was then pruned using a Python script to eliminate self-loop edges or duplicate edges. Phage gene similarity was visualized using Cytoscape v.3.8.2 (104). Different thresholds of edge weights (number of genes in common between phage genomes) were considered.

### Diversity of P. aeruginosa prophage integrase, C repressor, and terminase coding sequences.

The predicted phages were annotated using PATRIC v.3.6.9 with the Bacteriophage Domain, the Genetic Code for Bacteria and Archaea, and the Bacteriophage Annotation Recipe (98, 105). The resulting fasta descriptions for each gene contig were parsed to identify coding regions annotated as an integrase using the words “integrase” and “Integrase.” The same process was repeated to identify C repressor coding regions using the word “repressor” in combination with the word “cI,” “CI,” “c1,” or “C1.” Finally, the terminase coding regions were identified using the words “terminase” and “Terminase.” The annotated integrase, C repressor, and terminase coding regions were then compared for similarity to all other annotated coding regions from the predicted phages. A local BLAST (blastp) with a maximum of 1 target sequence was used with the non-integrase coding regions as the database and the integrase coding regions as the query sequences. Any gene that had a percent identity greater than 70% and a query coverage greater than 70% was then added to the respective list of integrases, and the same process was repeated for the C repressors and terminases. Each gene group was aligned using the MAFFT v.7.450 (106) multiple alignment with the automatic algorithm option with its default parameters. Using FastTree v.2.1.11 (107) with its default parameters, Newick trees were constructed and then visualized in iTOL v.6.1 (108). For easier visibility, clades with an average branch length less than 0.00056 were collapsed.

USEARCH v.11.0.667 (109) was used to create clusters of the integrase coding regions, clusters of the C repressor coding regions, and clusters of terminase coding regions. A threshold of “id = 0.7” was used for clustering. The clusters for the 3 genes were then uploaded to Geneious Prime 2020.1.2 to visualize the quality of the clusters. The phage memberships in the integrase, C repressor, and terminase clusters output from USEARCH were examined. A Python script using Biopython SeqIO, NumPy, and Pandas packages counted the number of phages shared between each of the 3 sets of coding region clusters (110–112). A one-sample *t* test was used to determine the statistical significance of the phage memberships. We examined if all members of one integrase/C repressor cluster shared the same terminase cluster and, thus, the same number of nonzero clusters.

### Predicting antibiotic resistance, virulence factors, and metabolic genes encoded by prophages.

ResFinder 4.1 (113) and its databases were installed and downloaded from the tool’s bitbucket page (https://bitbucket.org/genomicepidemiology/resfinder/src/master/). All of the predicted prophages were separately run using the authors’ suggestions of an 80% threshold and 60% minimum coverage for acquired antibiotic resistance genes. The virulence factors of the phages were then predicted using VFDB (114). The full data set that encompasses all virulence factor genes, both predicted and experimentally known, was downloaded from the VFDB website (http://www.mgc.ac.cn/cgi-bin/VFs/v5/main.cgi?func=VFanalyzer) and used as the BLAST database for local analysis via blastn with a threshold of greater than 70% identity. Each predicted phage was compared for sequence similarity to the virulence factor database entries.

To identify prophage coding regions associated with metabolism, the prophage FASTA format sequences were submitted to the MG-RAST webserver (115). No filtration of FASTA sequences was performed. Gene predictions assigned to a Metabolism COG category were retrieved from the webserver; these data include information about the prophage sequence containing the predicted gene, the COG number, the COG function, and the gene sequence. Python was used to parse this file to extract functional information.

### Mining for Pf1 phage sequences.

The Pf1 genome sequence (GenBank accession no. AY324828.1) was queried against the 5,383 P. aeruginosa genomes locally via blastn. Results were filtered, removing hits with a sequence identity or query coverage of <99%.
