# Supplementary material for: Diversity of Pseudomonas aeruginosa Temperate Phages
Source: mSphere. 2022 Feb 23;7(1):e01015-21. doi: 10.1128/msphere.01015-21 (PMC8865926; doi:10.1128/msphere.01015-21)
Supplement: FIG S1 [file msphere.01015-21-sf001.pdf]

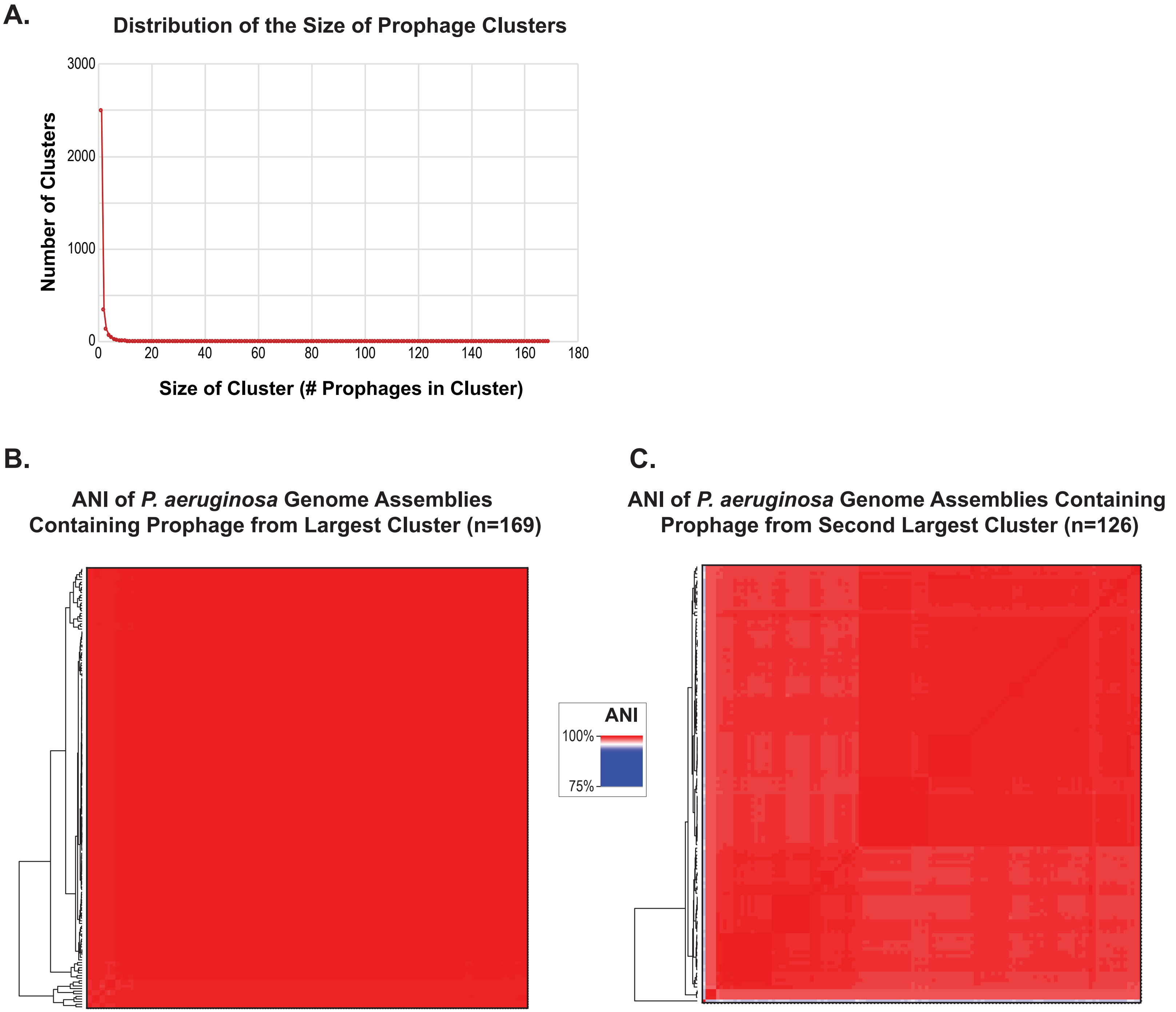

A.
Distribution of the Size of Prophage Clusters
Number of Clusters
3000
2000
1000
0
0
20
40
60
80
100
120
140
160
180
Size of Cluster (# Prophages in Cluster)
B.
ANI of P. aeruginosa Genome Assemblies Containing Prophage from Largest Cluster (n=169)
ANI
100%
75%
C.
ANI of P. aeruginosa Genome Assemblies Containing Prophage from Second Largest Cluster (n=126)
