# Supplementary figures and images for: Diversity of Pseudomonas aeruginosa Temperate Phages
Source: mSphere. 2022 Feb 23;7(1):e01015-21. doi: 10.1128/msphere.01015-21 (PMC8865926; doi:10.1128/msphere.01015-21)

Tree scale: 1

- Codes for an Integrase
- Codes for a C repressor
- Codes for Both

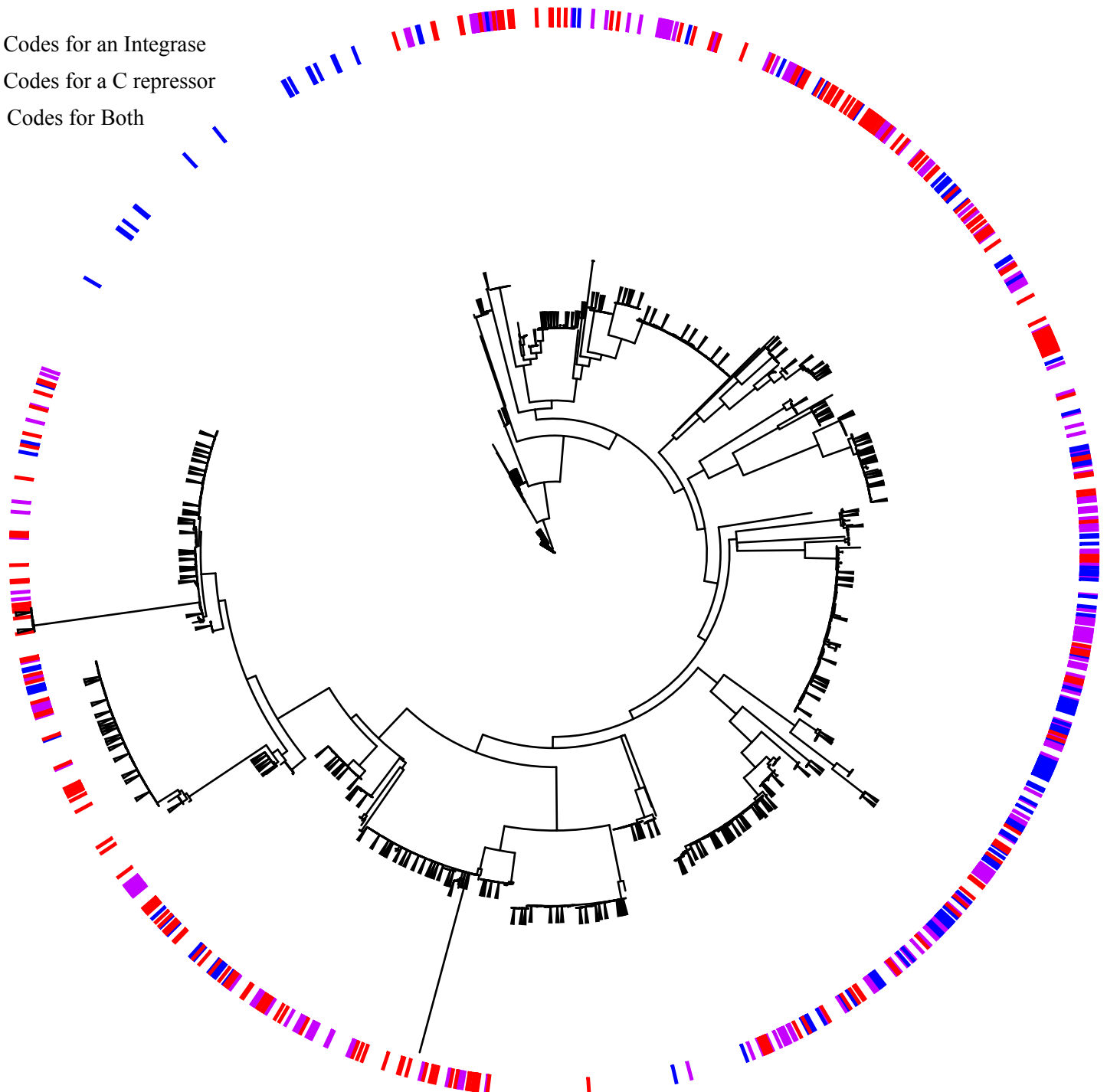

Supplement: FIG S2 [file msphere.01015-21-sf002.pdf]
